# Assessment of Multi-Image Unmanned Aerial Vehicle Based High-Throughput Field Phenotyping of Canopy Temperature

**DOI:** 10.3389/fpls.2020.00150

**Published:** 2020-02-25

**Authors:** Gregor Perich, Andreas Hund, Jonas Anderegg, Lukas Roth, Martin P. Boer, Achim Walter, Frank Liebisch, Helge Aasen

**Affiliations:** ^1^ Group of Crop Science, Department of Environmental Systems Science, Institute of Agricultural Sciences, ETH Zurich, Zurich, Switzerland; ^2^ Biometris, Wageningen University and Research Centre, Wageningen, Netherlands; ^3^ Water Protection and Substance Flows, Department Agroecology and Environment, Agroscope, Zürich, Switzerland

**Keywords:** thermography, unmanned aerial vehicle, phenotyping, plant breeding, spatial correction, low-altitude/high-resolution remote sensing, anisotropy, temperate climate

## Abstract

Canopy temperature (CT) has been related to water-use and yield formation in crops. However, constantly (e.g., sun illumination angle, ambient temperature) as well as rapidly (e.g., clouds) changing environmental conditions make it difficult to compare measurements taken even at short time intervals. This poses a great challenge for high-throughput field phenotyping (HTFP). The aim of this study was to i) set up a workflow for unmanned aerial vehicles (UAV) based HTFP of CT, ii) investigate different data processing procedures to combine information from multiple images into orthomosaics, iii) investigate the repeatability of the resulting CT by means of heritability, and iv) investigate the optimal timing for thermography measurements. Additionally, the approach was v) compared with other methods for HTFP of CT. The study was carried out in a winter wheat field trial with 354 genotypes planted in two replications in a temperate climate, where a UAV captured CT in a time series of 24 flights during 6 weeks of the grain-filling phase. Custom-made thermal ground control points enabled accurate georeferencing of the data. The generated thermal orthomosaics had a high spatial accuracy (mean ground sampling distance of 5.03 cm/pixel) and position accuracy [mean root-mean-square deviation (RMSE) = 4.79 cm] over all time points. An analysis on the impact of the measurement geometry revealed a gradient of apparent CT in parallel to the principle plane of the sun and a hotspot around nadir. Averaging information from all available images (and all measurement geometries) for an area of interest provided the best results by means of heritability. Correcting for spatial in-field heterogeneity as well as slight environmental changes during the measurements were performed with the R package SpATS. CT heritability ranged from 0.36 to 0.74. Highest heritability values were found in the early afternoon. Since senescence was found to influence the results, it is recommended to measure CT in wheat after flowering and before the onset of senescence. Overall, low-altitude and high-resolution remote sensing proved suitable to assess the CT of crop genotypes in a large number of small field plots as is required in crop breeding and variety testing experiments.

## Introduction

In view of current scenarios for climate change, canopy temperature (CT) is considered an important trait to select for adapted genotypes. CT was robustly associated with water status and stomatal conductance in wheat (Berliner et al., 1984; Blum et al., 1989; Amani et al., 1996). Low CTs have been associated with a 30% increased yield an increased water uptake by deeper roots (Lopes and Reynolds, 2010), when measured during grain filling. Even in regions with ample rainfall, such as the Swiss central plateau, heat and drought avoidance mechanisms connected to adjusted root system architecture may play an important role in extreme years (Oberholzer et al., 2017), projected to increase in frequency and severity in the near future. The regular assessment of CT during the breeding process holds great promise for an indirect selection of varieties with optimized rooting behaviour. A greater transpiration is a major driver leading to high yield potential of C3 crops under conditions characterized by low to moderate stress (Roche, 2015). It is, however, still a challenge to obtain reliable quantitative CT measurements for larger breeding experiments with small plots, since plot-by-plot CT measurements generally have a low repeatability (Pask et al., 2012; Rebetzke et al., 2013; Sukumaran et al., 2015; Deery et al., 2016) and are very time consuming.

The principle of elucidating plant evapotranspiration based on thermal remote sensing of CT has been used in a multitude of studies (Jones et al., 2009; Maes and Steppe, 2012; Liebisch et al., 2015; Khanal et al., 2017). It has been successfully applied to estimate grain yield (Elsayed et al., 2015; Becker and Schmidhalter, 2017; Elsayed et al., 2017), plant water, and plant drought stress (Calderón et al., 2013; Zarco-Tejada et al., 2013; Gómez-Candón et al., 2016), plant water status (Pou et al., 2014; Shafian and Maas, 2015; Bellvert et al., 2016), and soil water status (Hassan-Esfahani et al., 2015). unmanned aerial vehicle (UAV)-based thermography has been conducted in a multitude of studies as well (Zarco-Tejada et al., 2013; Gómez-Candón et al., 2016; Hoffmann et al., 2016; Ortega-Farías et al., 2016; Maes et al., 2017; Ribeiro-Gomes et al., 2017; Santesteban et al., 2017; Malbéteau et al., 2018; Sankaran et al., 2018; Sagan et al., 2019). Only a few studies (Liebisch et al., 2015; Deery et al., 2016; Rutkoski et al., 2016; Sagan et al., 2019), however, used it in a breeding context, where it would be strongly needed in the context of high-throughput field phenotyping (HTFP). HTFP aims for rapid and reliable assessment of phenotypic traits under field conditions. The lack of suited tools for HTFP has been identified as one of the main bottlenecks for plant breeding, slowing future breeding advances (Araus and Cairns, 2014; Walter et al., 2015). Moreover, a reliable method that allows repeated screening of a large number of plots in a short time period would be essential for thermal HTFP in particular, since the thermographic response of plants depends on the environmental conditions such as temperature, irradiance, and humidity that may change during the measurements.

Unmanned aerial vehicles (UAVs) are a low-altitude and high resolution remote sensing tool that promise to be an efficient carrier system for sensors used for vegetation monitoring (Anderson and Gaston, 2013; Colomina and Molina, 2014; Sanchez-Azofeifa et al., 2017; Aasen et al., 2018) including HTFP (Zaman-Allah et al., 2015; Deery et al., 2016; Hund et al., 2019). These carrier systems enable efficient data acquisition with a high spatial and temporal resolution at a relatively low cost (Berni et al., 2009b; Bellvert et al., 2016; Yousfi et al., 2016; Shakoor et al., 2017). Thus, they are also increasingly applied in field phenotyping applications (e.g., Gómez-Candón et al., 2016; Shakoor et al., 2017; Joalland et al., 2018; Sagan et al., 2019) and we hypothesize that they are also useful for HTFP of CT.

Two main approaches can be used to obtain remotely sensed plant CT form thermal imagery captured with airborne carrier systems at low altitude. The first approach is to take a single image to cover an area of interest (Zarco-Tejada et al., 2012; Calderón et al., 2013; Sankaran et al., 2018). The second approach is to mosaic multiple images together into an orthomosaic (Berni et al., 2009a; Gonzalez-Dugo et al., 2013; Hoffmann et al., 2016; Santesteban et al., 2017). The latter increases the area that can potentially be covered in one scene and thus, allows to capture larger areas and/or increase the spatial resolution (ground sampling distance, GSD) of the data by flying lower (Aasen et al., 2018). But when an orthomosaic is generated from multiple overlapping images, each area of interest on the ground (e.g., a plot) is captured by multiple images with different viewing geometries and several options exist to extract the signature of this area (Aasen and Bolten, 2018). It has been shown that the different data processing and extraction approaches have an influence on the apparent reflectance in remote sensing data in the visible and near-infrared, e.g., (Aasen, 2016; Aasen and Bolten, 2018). This results from the interaction of surface anisotropy (meaning that the signal is directionality dependent) and measurement geometry [expressed by the bi-directional reflectance distribution function; BRDF, (Nicodemus et al., 1977; Schaepman-Strub et al., 2006)], which also effects the apparent temperature (Jones et al., 2009; Cao et al., 2019). While studies have reported challenges when multiple thermal images are to be mosaicked together (Hoffmann et al., 2016; Ribeiro-Gomes et al., 2017), it has not been investigated how different data processing, mosaicking and capturing approaches affect the apparent CT.

Besides, the data may further include spatial trends due to field variability and temporal trends due to changes during the flight campaign. Correcting the data for these influences is essential if the genotypic performance is the major interest (Gilmour et al., 1997; Piepho and Williams, 2010; Araus and Cairns, 2014). Multiple approaches exist to perform this correction with the most widespread being first order autocorrelation models (Gilmour et al., 1997; Piepho and Williams, 2010) and P-splines (Velazco et al., 2017; Rodríguez-Álvarez et al., 2018), both implemented in a mixed-model framework.

This study combines the above mentioned aspects and aims for an integrated concept how CT can be assessed from high-resolution UAV remote sensing within a breeding context. In particular, the study aims to:

- Establish a workflow for high-resolution UAV remote sensing HTFP
- Investigate and discuss different data processing modes to generate thermal orthomosaics
- Evaluate the method in a temperate environment across the season
- Investigate the optimal timing for thermography measurements
- Compare the UAV approach to other established approaches for HTFP of CT

## Materials and Methods

### Experimental Site and Wheat Cultivation

**Figure 1:**
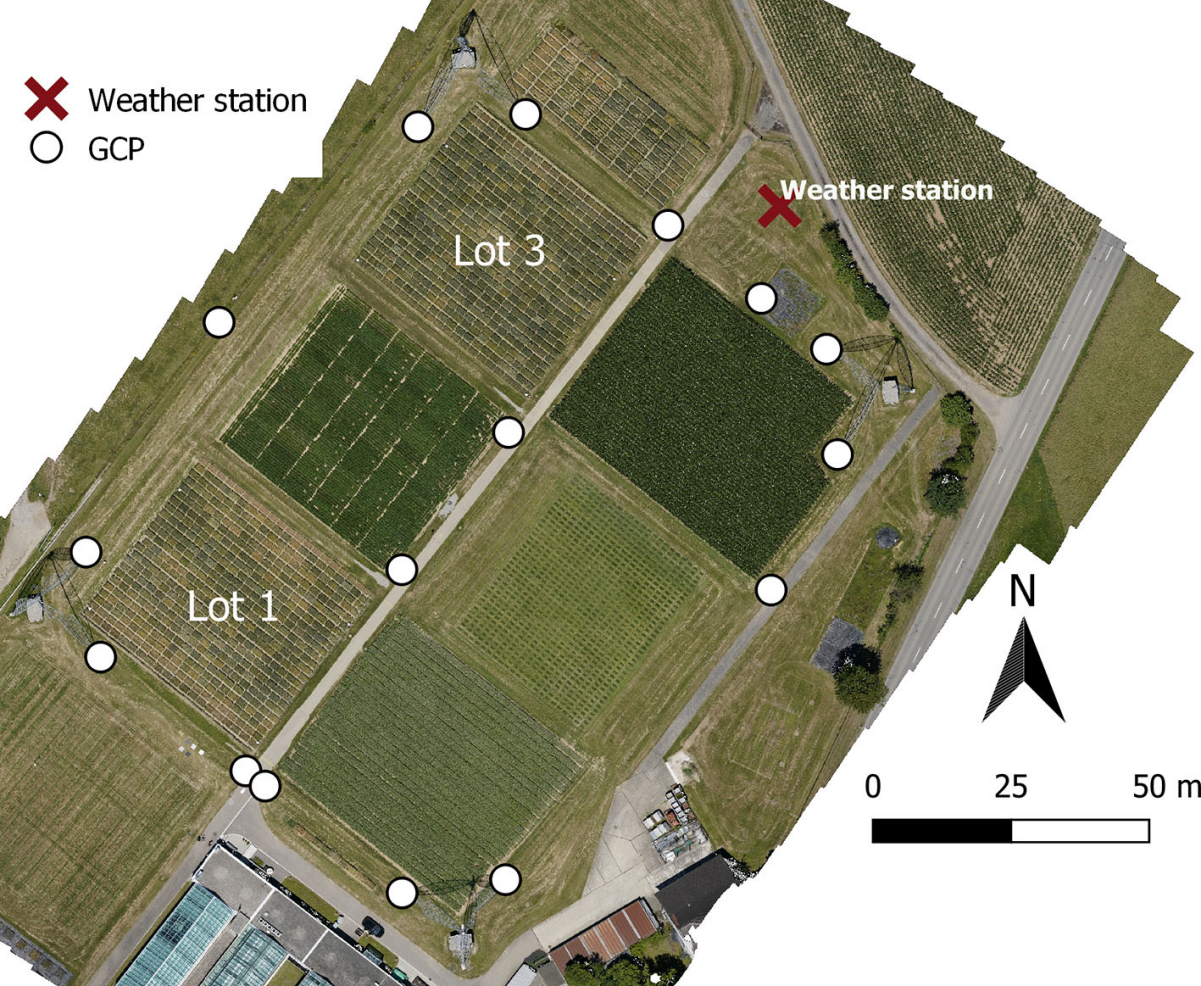
Red green blue (RGB) orthophoto of the “FIP” field at the agricultural research station in Eschikon with the location of the 16 ground control points (GCPs) (white dots), the two replicates of the winter wheat field trial (lot 1 and lot 3) and the weather station (red cross).

A field experiment was conducted at the ETH field phenotyping platform field phenotyping platform (FIP) (Kirchgessner et al., 2017), a one-hectare field (“FIP field”) located at ETH Zurich's plant research station [47°27′01″N and 8°40′57″E, the World Geodetic System (WGS) 84]. The soil type is a skeleton rich variable Cambisol (stagnic to slightly acidic appearance) with 21% clay, 21% silt, and 3.5% organic matter. The “FIP field” employs a crop rotation containing major crops of Switzerland's agricultural system in six lots (**Figure 1**). The experiment was sown in two replicates (represented by lots one and three in the “FIP field,” **Figure 1**) and consisted of 354 winter wheat genotypes, mainly from the GABI-wheat panel (Kollers et al., 2013) with additional Swiss varieties. Three out of the 354 genotypes were used as checks (CH Claro, Suretta, and CH Nara). The genotypes were distributed to the experiment using the R package “DiGGer” (Coombes, 2009) in an augmented 2D design as follows: The check varieties were distributed within each replication (**Figure 1**) in nine complete blocks (seven rows by six ranges), making sure that at least one check was placed in each row and each range of the design (27 check plots per replication in total). Onto this design, the 351 test genotypes were augmented to incomplete blocks in row (one row per incomplete block) and range direction (6 ranges per incomplete block). The 351 test genotypes and the 27 check plots resulted in a total of 378 plots for each replication or 756 plots for the whole experiment. Each replication of the experiment consisted of 21 ranges and 18 rows. For the growing season 2018, winter wheat was sown on both replicates on the 2017-10-17 with a sowing density of 400 seeds per m^2^. The size of the winter wheat replications was approximately 40 m x 36 m. The individual plots had a length of 1.7 m and a width of 1 m with a row spacing of 12.5 cm equating to nine rows per plot. Harvest of the winter wheat was on the 2018-07-13. Weather data was obtained by the on-site weather station (**Figure 1**). 2018 was a very dry summer with no rain between the 2018-06-14 and 2017-07-02 (for details, see section *Canopy Temperature Heritability Across a Day and Dates*). During the whole season BBCH growth stages (Lancashire et al., 1991) were rated in the field. Canopy senescence was scored visually in 2–3 day intervals by estimating the overall greenness of the plot when inspected at a viewing angle of approximately 45°. An integer mean value per plot was estimated on a scale ranging from 0 (completely green canopy) to 10 (completely senescent canopy). The onset of senescence was defined when a plot reached a scoring of greater than zero. The first genotypes started to become senescent on the 2018-06-16. Based on these measurements we defined a set of 178 genotypes that showed no sign of visual senescence up to the 2018-06-23, the “stay green” genotypes (see sections *Canopy Temperature Heritability Across a Day and Dates* and *Canopy Temperature Correlation Across a Day and Dates*). After that date, the set of “stay green” genotypes became very small.

### Unmanned Aerial Vehicles Flights

Twenty-four UAV flights between early June 2018 and mid-July 2018 were performed covering the grain filling and ripening phase (BBCH growth stages 73-92). Most flights were carried out around solar noon or in the early afternoon and under stable cloud cover (no clouds or a sparse cloud cover). On the 2018-06-16 and the 2018-06-20, multiple flights from the morning to the late afternoon were carried out. On the 2018-06-16 cloud conditions were fluctuating, with the photosynthetically active radiation (PAR, measured in photosynthetically active photon flux density) fluctuating between 750 and 2,200 μmol m^−2^s^−1^. On the 2018-06-20 the conditions were very stable with a typical diurnal cycle of temperature and PAR. Thus this day is referred to as “stable day.” The flight dates and corresponding BBCH stages can be found in **Table 1** of the **Supplementary Materials**. Geo-referencing the thermal scenes (section *Processing of Thermal Data*) was done using thermal ground control points (GCPs). These custom-made GCPs consisted of a styrofoam plate of dimensions 0.5 m x 0.5 m x 0.04 m glued onto a wooden panel. On top of the styrofoam panel, two black aluminum triangles were glued to obtain a distinctive cross-shaped GCP (for details see section *Processing of Thermal Data*). The black aluminum plates heated up considerably more than the white Styrofoam, showing as a distinct pattern in the GCP. Sixteen GCPs were evenly distributed across the experimental site (**Figure 1**) and their positions were measured using a Trimble R10 GNSS (Global Navigation Satellite System) receiver (Trimble Ltd., USA) with swipos-GIS/GEO RTK (Real Time Kinematic) correction (Federal Office of Topography Swisstopo, Wabern, Switzerland) with an overall horizontal and vertical precision of 0.1 m.

#### Unmanned Aerial Vehicles Platform

The UAV platform was a DJI Matrice 600 Pro (SZ DJI Technology Co. Ltd., China). The total weight of the UAV, including the batteries, is 9.5 kg, leaving a maximum payload of 6 kg. The UAV uses a DJI A3 flight controller, which was upgraded to A3 Pro standard with an enhanced GNSS system for position data. The UAV was controlled using the DJI Matrice 600 series remote controller and an iPad (Apple Inc., USA) with the DJI Ground Station Pro app (SZ DJI Technology Co. Ltd., China). The UAV requires six charged 99.9 Wh batteries for operation. With the payload, flight times are about 15 min.

#### Thermal Camera System

**Figure 2:**
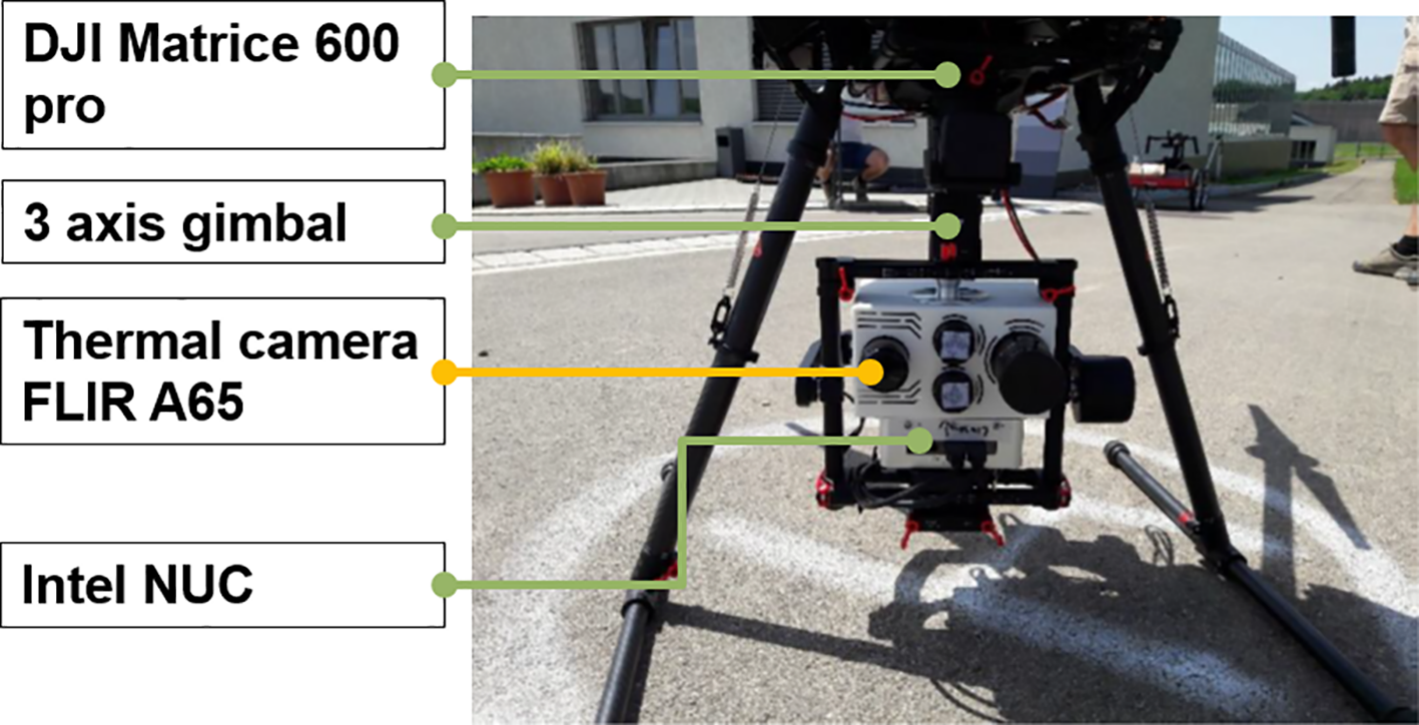
The sensor package (white box) with the four cameras and the Intel NUC computer mounted on the gimbal beneath the unmanned aerial vehicle (UAV). All cameras still have their protective caps on.

A radiometrically calibrated FLIR A65 thermal imaging camera (FLIR integrated Imaging Solutions Inc., Canada) was mounted in a custom-made sensor package (**Figure 2**). The thermal camera has a field of view (FOV) of 25° x 20° and a resolution of 640 x 512 pixels. The camera's sensor is an uncooled Vanadium Oxide (VOx) microbolometer detector with a detector pitch of 17 µm measuring in the spectral range of 7.5–13 µm. The maximum image frequency of the camera is 30 Hz. It weighs approximately 0.2 kg and is connected to an Intel^®^ NUC computer through a standard RJ45 LAN cable. The specified temperature range of the measurement objects is −40°C to +550°C. The noise equivalent temperature difference (NETD) of the camera is 0.05°C at 30°C and the absolute measurement accuracy is ±5°C or 5% of the readings (FLIR Systems, 2014). The camera system was controlled by a self-developed MATLAB (MATLAB R2017b, The MathWorks Inc. USA) script running on a compact Intel^®^ NUC computer (i7-5557U dual core processor, 16GB RAM and a 256GB SSD, Windows 10 operating system). The whole system was mounted on a three-axis stabilized DJI Ronin-MX Gimbal (SZ DJI Technology Co. Ltd., China), to ensure a nadir viewing geometry (**Figure 2**).

#### Measurement Protocol

Mission planning was conducted in the “PhenoFly Planning Tool” (Roth et al., 2018). The flight details were: 80 m height above ground level with an image overlap of >70% across to flight direction and >90% in the along direction. Images were acquired at a rate of 2.2 Hz. Average flight duration was 8 min to cover the two replications (lots one and three, **Figure 1**). Since uncooled thermal cameras tend to drift when their temperature change (Mesas-Carrascosa et al., 2018; Kelly et al., 2019), the camera was turned on more than 30 min before the measurements [as recommended by Berni et al. (2009b) and Kelly et al. (2019)] to allow temperature stabilization of the system. After take-off at the instant before the measurement sequence was started, a non-uniformity correction (NUC) was manually triggered. No further NUCs were performed during the flight since during the time of the flight, the temperature of the sensor did barely change (chip: ~0.2°C, housing: ~0.4°C according to the sensor metadata).

### Processing of Thermal Data

#### Photogrammetric Processing

**Figure 3:**
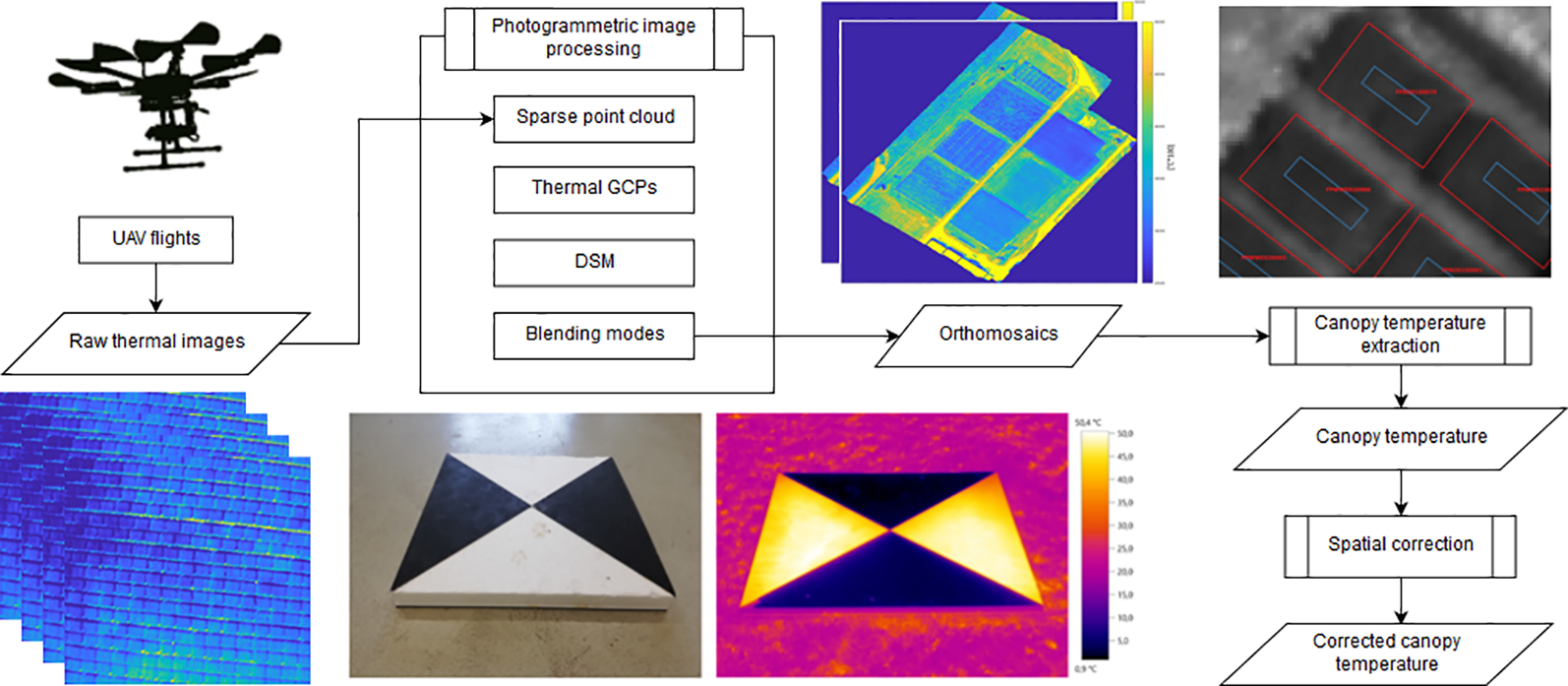
Schematic summary of the workflow for obtaining high-accuracy thermal orthomosaics from single, raw thermal unmanned aerial vehicles (UAV) images. The thermal ground control points (GCPs) used in this study are depicted in the bottom center: as seen in red green blue (RGB) (left) and through a handheld infrared (IR) camera. The canopy temperature (CT) extraction through the polygons representing the plots is shown top right.

The processing of thermal data is summarized in **Figure 3**. After the raw data (raw digital numbers, DN) of each image was converted to °C, photogrammetric data processing of the thermal images was done in Agisoft PhotoScan Professional 1.4.3 (Agisoft LLC, St. Petersburg, Russia). Agisoft PhotoScan is a software performing the Structure from Motion (SfM) algorithm, which enables capture of the 3D structure of objects by a 2D transformation of a set of their projected images (Ullman, 1979). It allows derivation of 3D information through exploitation of feature points found in overlapping images (Harwin and Lucieer, 2012). SfM performs image matching by calculating the relative position of a series of images by identification of feature points. The feature points are used in bundle adjustment, which estimates viewing parameters (camera positions and/or calibration) estimates for the individual images (Triggs et al., 2000). Bundle adjustment results in a set of 3D points, corresponding to a sparse 3D point cloud. The “image alignment” in Agisoft PhotoScan was run using quality parameter set to “high,” a key point limit of 40,000 and a tie point limit of 1,000. Additionally, pre-estimated camera parameters were loaded and set to fixed to ensure a consistent generation of the orthomosaics. The quality setting “high” compressed the image quality by half (Agisoft LLC, 2016) but greatly reduced processing times. The point clouds were georeferenced to the coordinate system EPSG:2056 (CH1903+/LV95) using the thermal GCPs (**Figure 3**, bottom center). The GCPs were manually marked in three to four images for each GCP, until the algorithm picked up their correct locations across all images. This referencing also optimized the sparse point cloud, correcting distortion effects. The density of the optimized sparse point cloud was increased in the “build dense cloud” step in Agisoft PhotoScan, resulting in a dense point cloud. The “build dense cloud” was performed using the “high” quality and “aggressive” depth filtering settings. The georeferenced dense point cloud was then used to generate a digital surface model (DSM), effectively representing the captured surface in three coordinates.

#### Generation of Orthomosaics

The georeferenced DSM was then used to generate a thermal orthomosaic of the UAV flights through mosaicking of the individual images (“Orthomosaic” is hereafter used interchangeably with “thermal orthomosaic” in this study, unless stated otherwise). Agisoft PhotoScan offers multiple processing modes to calculate an orthomosaic, of which the following two were chosen in this study:

- In the blending mode “average,” the values of all pixels from all images that covered a point in the orthomosaic were averaged. Consequently, each pixel in the final orthomosaic originate only from many images.
- In the blending mode “disabled,” the pixel value from the image with a view being closest to the normal at that point (nadir) was used. Consequently, each pixel in the final orthomosaic originate only from one image.

Consequently, the angular properties of the data within the two different types of orthomosaics differ. The viewing geometry of each pixel in the blending mode “disabled” orthomosaic is the same as in the original image and thus, very narrow (because of the narrow instantaneous field of view of every pixel) and can be described as (almost) directional measurement geometry (Schaepman et al., 2015; Aasen and Bolten, 2018). In the blending mode “average,” the viewing geometry is composed by all the viewing geometries of the pixels that are averaged for one pixel in the orthomosaic. Thus, the total viewing geometry of each pixel in the orthomosaic is wider than in the blending mode “disabled” and can be described as conical measurement geometry (Schaepman et al., 2015; Aasen and Bolten, 2018). While a detailed description and discussion on these differences for spectral data can be found in Aasen and Bolten (2018), this paper will investigate the effects on the apparent CT within orthomosaics. This will be done by qualitatively comparing the two blending modes for the flight on the 2018-06-20 at 14:00 h and quantitatively investigating the viewing geometry dependency of the apparent CT on the same date. Additionally, we used a Bland-Altman analysis (Bland and Altman, 1986) to estimate systematic differences between the two blending modes in relationship to the plot mean temperature. Differences in the heritability (section *Spatial Correction and Heritability Calculation*) will also be investigated.

#### Plot Wise Canopy Temperature Extraction and Normalization

To extract the per-plot UAV temperature, a polygon describing the plot shape and location was generated using the experimental design. QGIS 3.2.3 Geographic Information System Software (QGIS Development Team, 2018) was used to create an inward buffer of 50 cm from the shapes to omit edge effects (**Figure 3**, top right: blue polygon). Based on a Python 3.6 script, the median of this area was then used as CT for a plot. The CT was normalized by the ambient air temperature (T_A_) to compare temperatures across different measurement dates (Balota et al., 2007; Maes and Steppe, 2012; Zarco-Tejada et al., 2013; Bellvert et al., 2016) as follows:

(1) $$\Delta T=T_{C}-T_{A}$$

*T_A_* was measured at 2 m above ground level by a temperature sensor (CS215, Campbell Scientific, Inc., USA) covered by a 10-Plate Solar Radiation Shield (RAD10, Campbell Scientific, Inc., USA) situated in the on-site weather station (**Figure 1**).

### Spatial Correction and Heritability Calculation

The correction of spatial trends as a result of both, spatial variability of trait (CT) values in the field and, in case of CT, additional changes during the flight campaign was done with the R-packages SpATS (Rodríguez-Álvarez et al., 2018). For each UAV flight, a model was fitted with a peculiarity of the experimental site in mind: generally we observe a strong pattern in the replications (lots one and three, **Figure 1**) in working direction (row direction) while there are more smooth trends perpendicular to this direction (range direction). The spatial model was:

(2) $$Y=f(r,c)+Z_{g}c_{g}+Z_{r}c_{r}+\varepsilon$$

where *f* (*r*,*c*) is a smoothed bivariate surface defined over row (r= 1–74) and range (c=1–18) positions of a virtual grid in which both replication were arranged (see below). The vector c_g_ = (c_g1_, …, c_g354_) is the random coefficient of the genotypes associated with the design matrix Z_g_, c_r_ = (c_r1,_ …, c_r74_) ~ N(0, σ_r_^2^I_74_) is the random coefficient of the rows associated with design matrix Z_r_ and ε is the random error vector ε= (ε_1_, …,ε_n_) ~ N(0, σ^2^I_n_). Replication 1 (lot 1) ranged from row 1 to 21 and range 1 to 18 while replication 2 (lot 3) ranged from row 54 to 74 and range 1 to 18 in the virtual grid. Thus, there were 32 rows separating the two replications in the virtual grid representing lot two (the parcel between lots one and three, **Figure 1**). The number of spline points was set to 2/3 of the total number of rows and ranges in the virtual grid, respectively. To calculate the best linear unbiased estimator (BLUEs), the genotypes were set as fixed-effects and the design matrix in equation two became X_g_ accordingly. The spatially corrected plot values were derived as the sum of model intercept, plot-specific genotypic BLUEs and residual error. Heritability of the spatially corrected traits (model two) was calculated according to (Rodríguez-Álvarez et al., 2018) based on the genetic effective dimensions provided by SpATS as:

(3) $$H_{s}^{2}=\frac{ED_{g}}{n_{g}-1}$$

where ED_g_ is the effective dimension for the genotypes and n_g_ is the total number of genotypes evaluated. The denominator (n_g_–1) reflects the upper bound for the effective dimension [see Rodríguez-Álvarez et al. (2018) for further details].

## Results

### Analysis of Orthomosaics Resulting From Different Blending Modes

**Figure 4:**
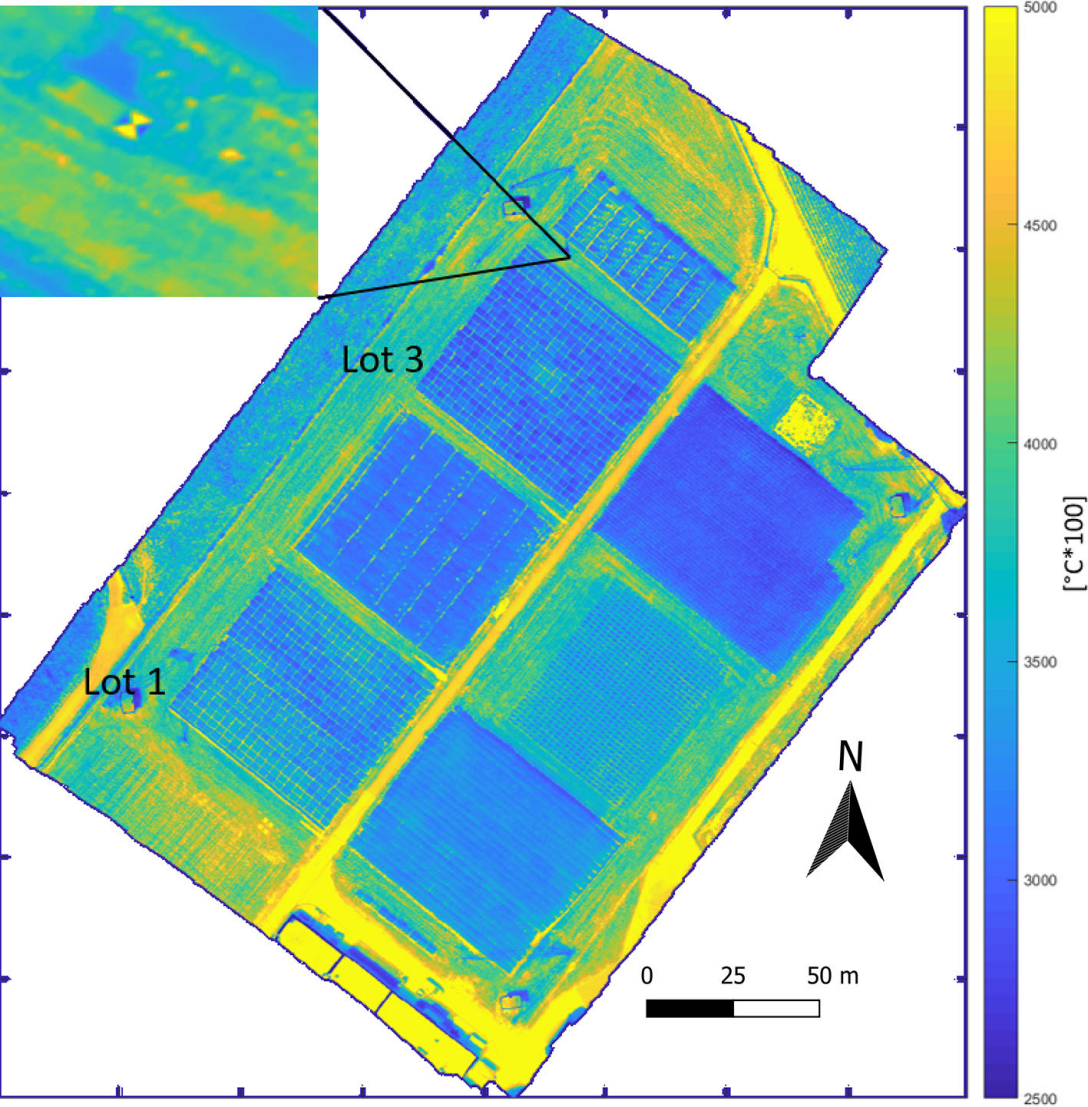
Thermal orthomosaic of the experimental site [the “field phenotyping platform (FIP) field”] mosaicked using the blending mode “average” from the flight on the 2018-06-23 at 15:09 h local time. The orthomosaic has a dimension of 3,990 x 4,490 px, a ground sampling distance of 4.89 cm/px at a flight height of 87 m (estimated by Agisoft PhotoScan). The enlarged area (top left) shows a thermal ground control point (GCP) as seen in the orthomosaic. Note the “chessboard”-like structure of the individual wheat plots in the two replicates (lots one and three).

Processing the thermal data (section *Processing of Thermal Data*, **Figure 3**) resulted in orthomosaics such as shown in **Figure 4**. The thermal GCPs (section *Unmanned Aerial Vehicles Flights* and **Figure 3**) were clearly visible in the orthomosaic (**Figure 4**), leading to an overall high spatial accuracy. The obtained GSD of these orthomosaics varied from 4.89 to 5.11 cm due to slight variations in flying altitudes. The calculated GSD of the thermal camera used at a flight height of 80 m was 5.5 cm. The root-mean-square deviation (RMSE) of the GCP positions across all 24 UAV flights ranged from 1.25 to 10.05 cm with a mean RMSE of 4.79 cm. The exact accuracy metrics for each flight date can be found in **Table 2** of the **Supplementary Materials**.

**Figure 5:**
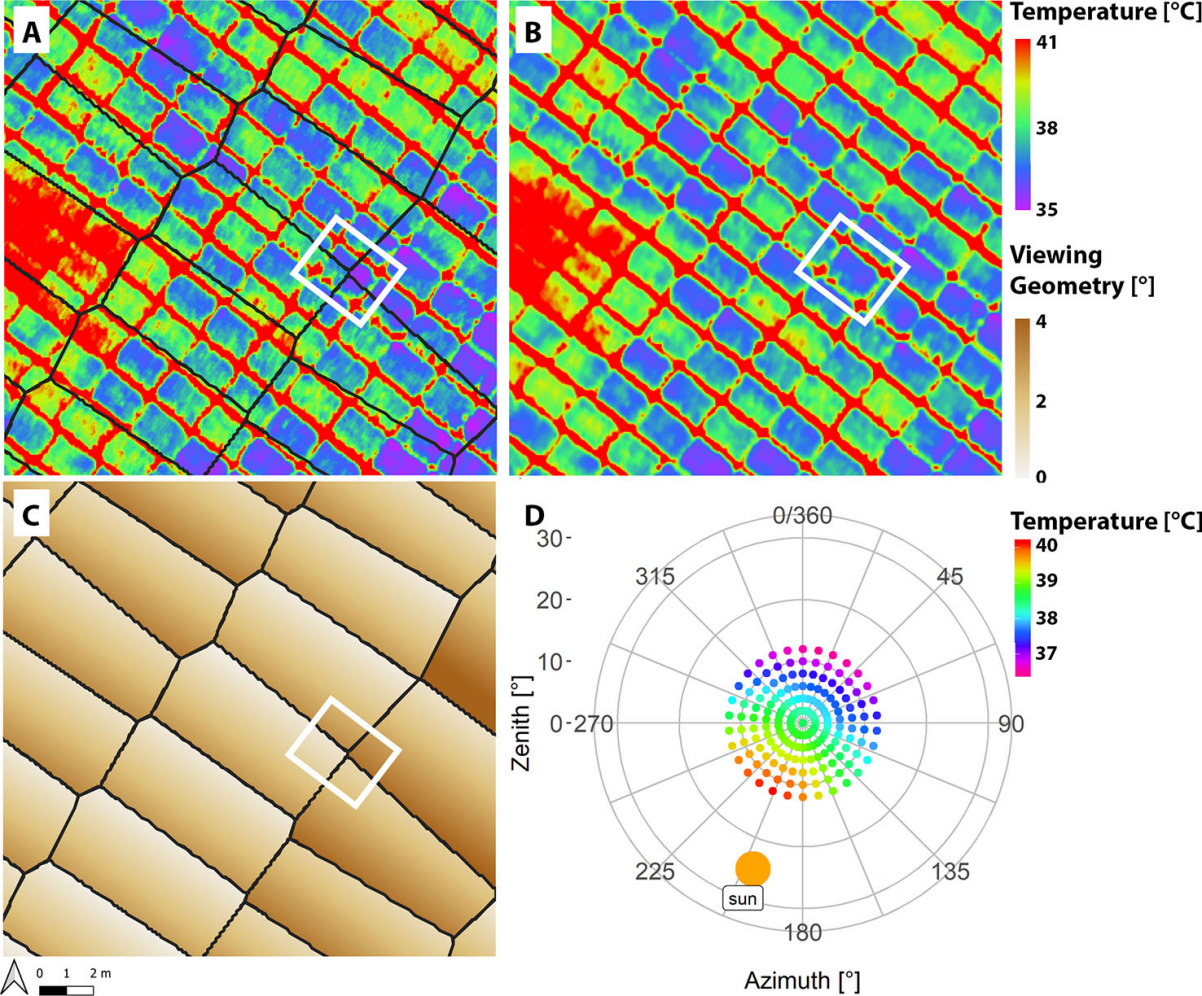
Excerpt of an orthomosaic generated with blending mode “disabled” **(A)** and “average” **(B)** on the 2018-06-20 at 14:00 h local time. The color corresponds to the apparent canopy temperature (CT). **(C)** shows the viewing geometry of the information used to generate the blending mode “disabled” orthomosaic **(A)**. In **A** and **C**, black seamlines mark the border of the information that is taken from different images. The white box highlights a plot where information from four images are composed in the blending mode “disabled” mosaic **(A)**. **(D)** shows the average apparent temperature of all plots of that flight in dependence of its viewing geometry. The sun had an azimuth angle of approx. 199° and a zenith angle of approx. 25°. The viewing geometry of the apparent temperature was calculated from the relative position of the camera seen from the plot.

A detailed look at thermal orthomosaics revealed that the viewing geometry influenced the apparent CT. **Figure 5** exemplifies the situations for the flight on the 2018-06-20 at 14:00 h. **Figure 5A, B** show the orthomosaics generated with the blending mode “disabled” and “average,” respectively. The hot areas are the paths between the plots. The high spatial resolution reveal differences within the plots of up to several °C. Approx. 20 cm (four pixels) within every plot seem to be influenced by border effects in both orthomosaics. A qualitative comparison of the orthomosaics showed more apparent heterogeneity in “disabled” mode.

The black lines in **Figure 5A, C** denote seamlines between information of the different images used to compose the orthomosaic in blending mode “disabled.” The white rectangle highlights a plot that is composed by information from four different images. Within the plot, the apparent temperature changes along the seamlines. **Figure 5C** shows that, at this point, information with different viewing geometries (about 4° difference) have been composed next to each other. For a detailed explanation and schematically drawing on how an orthomosaic is composed please refer to Aasen and Bolten (2018), and for a detailed description on how to trace pixel dependent properties please refer to Aasen et al. (2015). Generally, only very small ranges of viewing geometries are used in the blending mode “disabled.” In the blending mode “average” (**Figure 5B**), sharp transitions between apparent temperatures are not visible. In this mode the information of more than 20 images was averaged and thus, a wide range of viewing geometries were used.

**Figure 5D** shows the average apparent temperature of all plots of that fight in dependence of its viewing geometry. The sun had an azimuth angle of approx. 199° and a zenith angle of approx. 25° (retrieved from https://www.suncalc.org, for Lindau, Zurich, CH at 14:01 h UTC+2). The viewing geometry of the apparent temperature was calculated from the relative position of the camera seen from the plot. Thus, a viewing geometry of 0° azimuth and zenith corresponded to nadir (measurement right above the plot), and a viewing geometry of 20° zenith and 199° azimuth would have an acute angle while 20° zenith and 19° azimuth would have an obtuse angle to the sun. The plot reveals that, on average, the apparent temperature differs by more than 3.5°C (36.5–40.1°C) within the different viewing geometries within an image, with the largest gradient in direction of the principal plane of the sun were the measurement geometry (sun-object-sensor) changes from an obtuse angle to an acute angle. A close look at **Figure 5D** reveals that around nadir—where the proportion of soil signal is higher compared to other viewing geometries—the temperatures are slightly increased when compared to the general pattern. High-resolution thermal imagery captured by the ETH field phenotyping station (Kirchgessner et al., 2017) explains this observation, since in-between the crop rows the warm soil can be seen (see **Figure 1** in the **Supplementary Materials** for an example).

**Figure 6:**
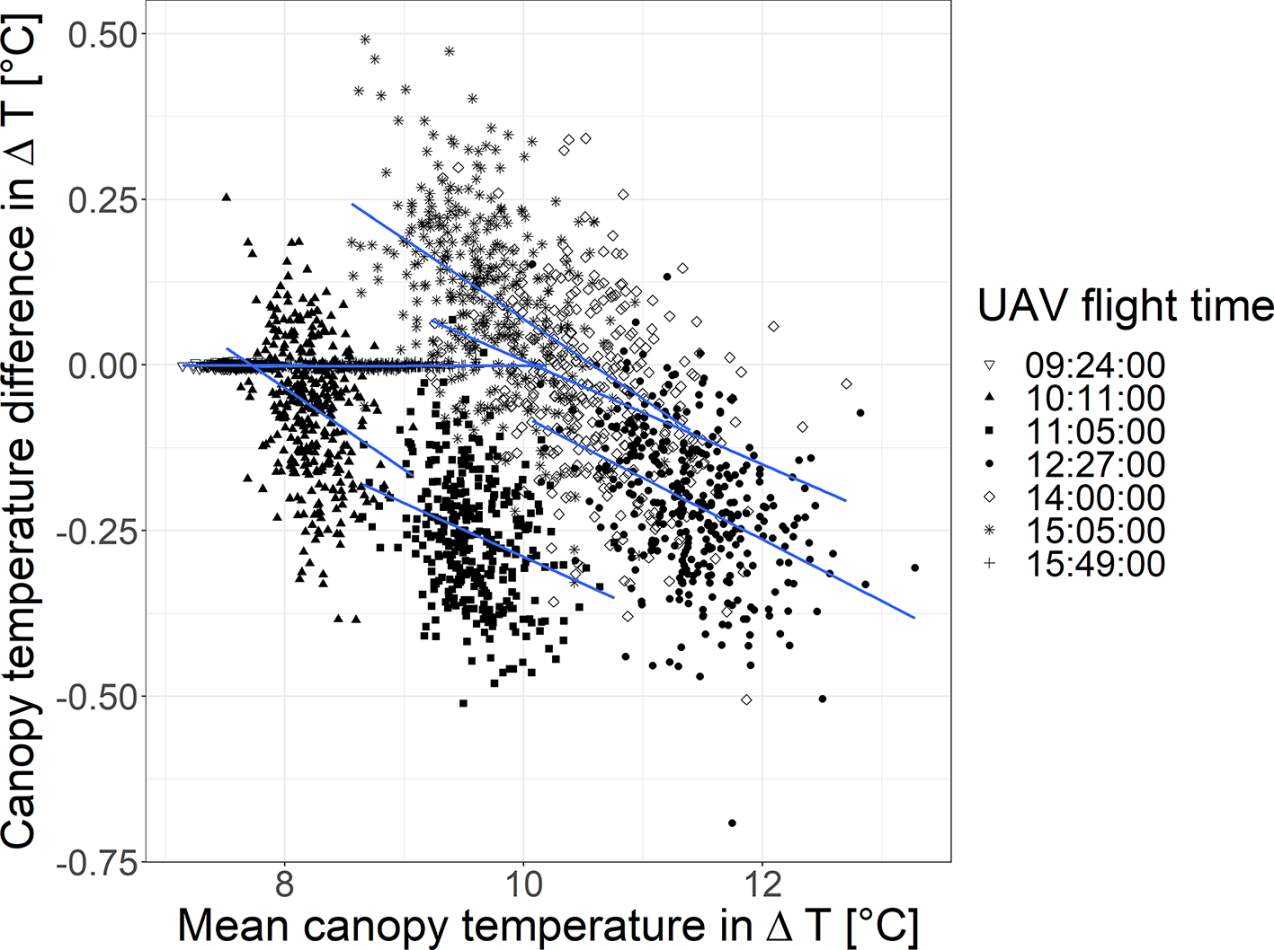
Bland-Altman plot showing the mean canopy temperature (CT, in ΔT) of both blending modes (“average” and “disabled”) on the x axis and the CT difference (in ΔT) between both blending modes on the y axis (“average” minus “disabled”) for measurements taken on the 2018-06-20.

For all flights together, the CT values obtained by the two evaluated blending modes were linearly related across all UAV flights (intercept = 0.14; slope = 0.96; R^2^ = 0.98, **Figure 2** in the **Supplementary Materials** together with linear relationships for each UAV flight). **Figure 6** shows a Bland-Altman plot of the 2018-06-20 containing the relationship of the plots mean CT across both blending modes (x axis) to the difference in CT between both blending modes (y axis, “disabled” subtracted from “average”). It allows comparing systematic differences between the two blending modes. Overall, the difference became more negative until noon and increased toward the late afternoon. For the first and last flight, the difference between the blending modes was negligible. All other UAV flights exhibited a slightly negative trend between the mean CT and the CT difference between the blending modes.

### Canopy Temperature Heritability Across a Day and Dates

**Figure 7:**
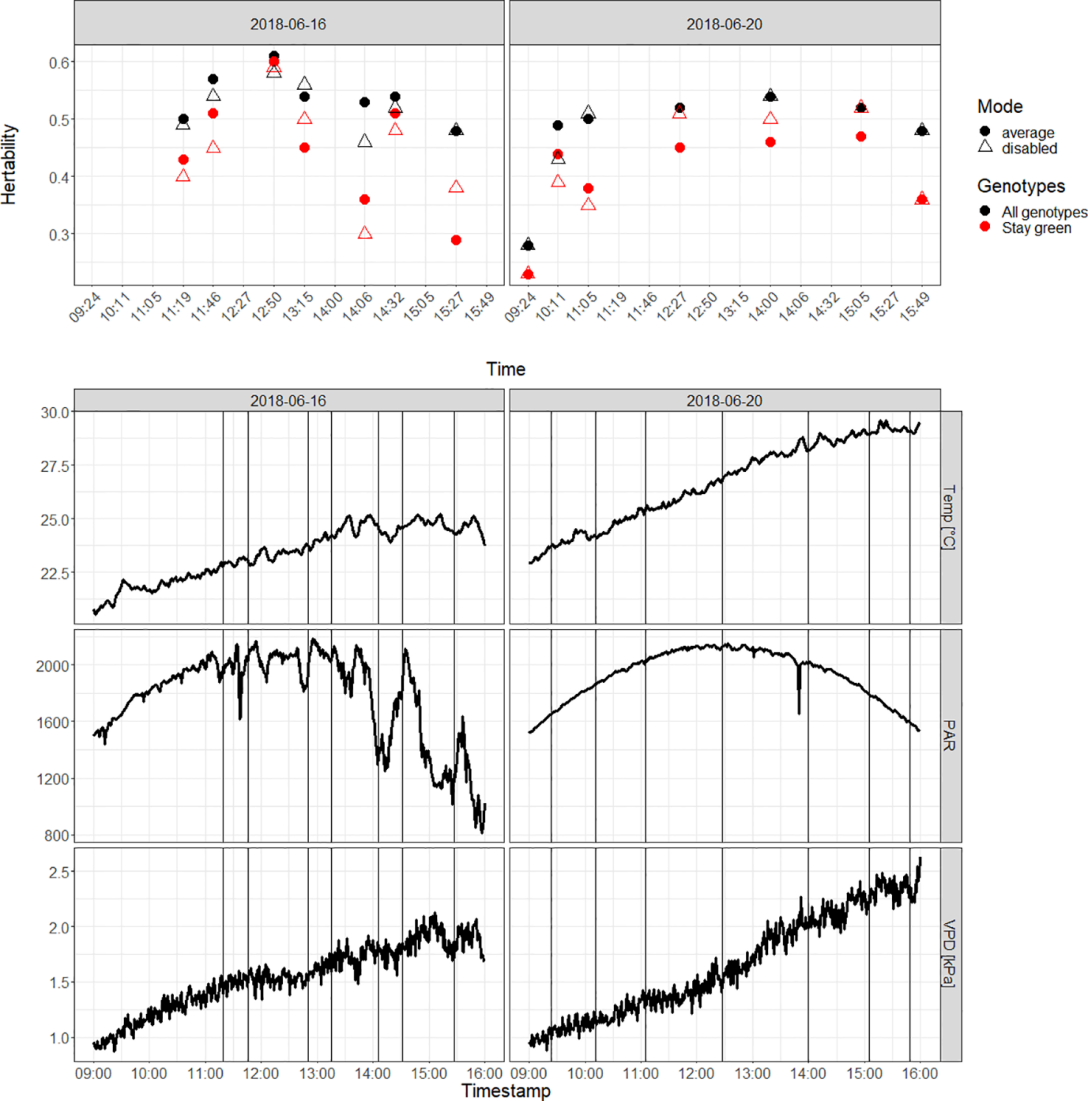
Diurnal variation of H2 values on the 2018-06-16 and the 2018-06-20 (top) for all genotypes (black) and for “stay green” genotypes (red). Data were gathered using the blending mode set to “average.” Axis times in hours:minutes, local time. Diurnal variation of the temperature, the photosynthetically active radiation (PAR in μmol m^−2^s^−1^) and the vapor pressure deficit (VPD) on the 2018-06-16 and the 2018-06-20 (bottom). The vertical lines correspond to the start time of the unmanned aerial vehicles (UAV) flights.

For the first day with multiple flights—the 2018-06-16—fluctuations in PAR due to cloud passes and, to a lesser extent, vapor pressure deficit (VPD) (**Figure 7**, bottom) resulted in variable H^2^ values (**Figure 7**, top). For all genotypes, H^2^ values ranged from 0.46 to 0.58 for the blending mode “disabled” and from 0.48 to 0.61 for the blending mode “average.” Overall, H^2^ increased during the morning, peaking at 12:50 h on the 2018-06-16, right after a passing of clouds. H^2^ values of the “stay green” genotypes were low at the 14:06 h and the 15:27 h measurements. They ranged from 0.29 to 0.6 for the blending mode “average” and from 0.3 to 0.59 for the blending mode “disabled.”

For the second day with multiple flights, the 2018-06-20, weather conditions were stable and the H^2^ values were similar for most flights (**Figure 7**, top). The H^2^ values of the 9:24 h measurement were low with values under 0.3 for both blending modes and sets of genotypes. The other UAV flights showed higher H^2^ values ranging from 0.48 to 0.54 for the blending mode “average” and from 0.43 to to 0.54 for the blending mode “disabled.” On that day, highest H^2^ was reached at 14:00 h before decreasing again. The “stay green” genotypes exhibited lower H^2^ values than all genotypes throughout the 2018-06-20 with the exception of the blending mode “disabled” measurement at 15:05 h. The pattern of H^2^ values for these genotypes was similar to the one found in all genotypes.

**Figure 8:**
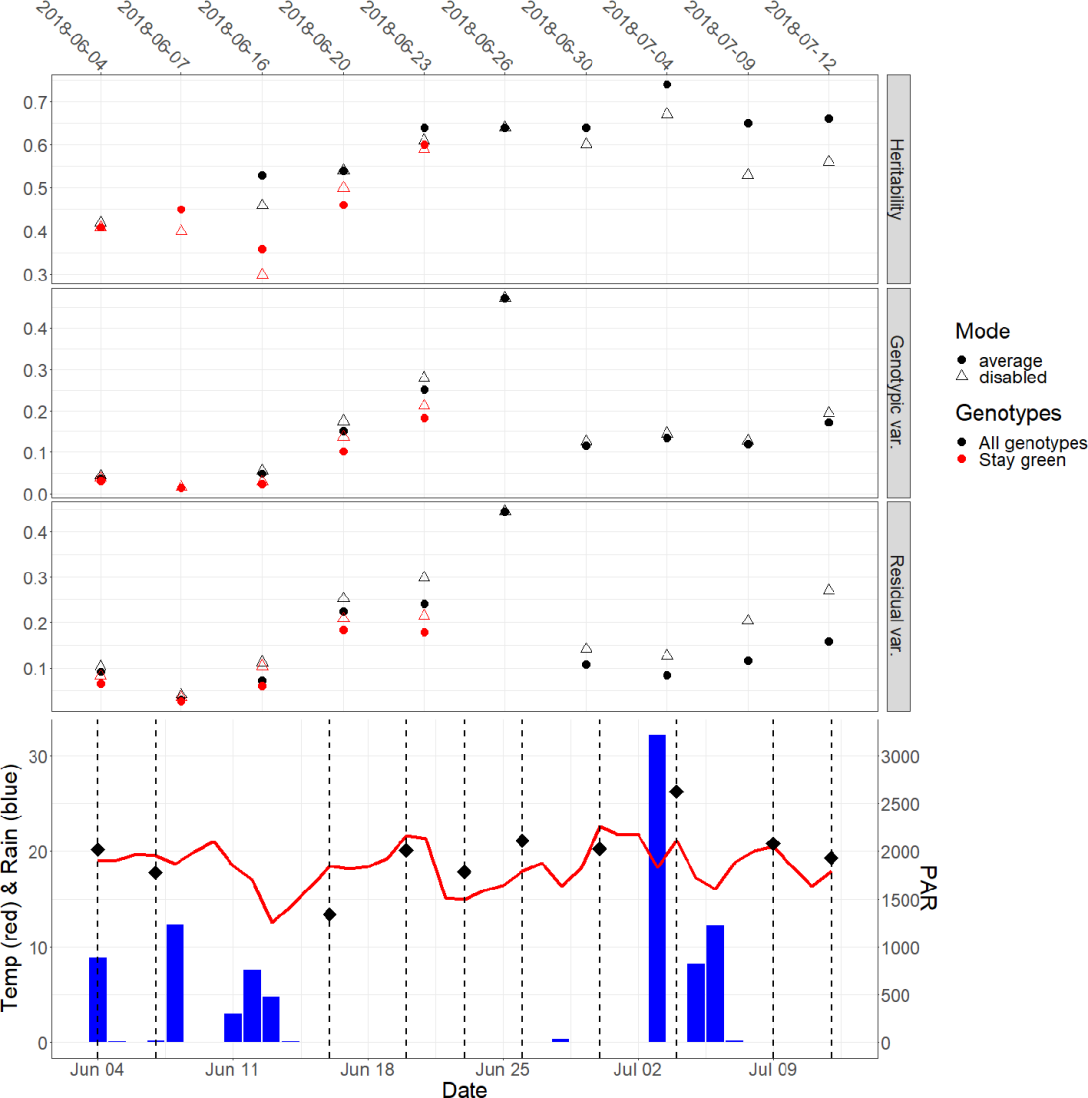
Heritabilities for the solar noon measurements shown for spatially corrected data (top) for all genotypes (black) and for “stay green” genotypes (red). The genotypic and residual variances of the two blending modes are also plotted (middle panels). The genotypic and the residual variance of the SpATS corrected data was overall lower for the blending mode “average.” Weather data (bottom) is given in mean daily air temperature (°C, red line) and cumulated daily precipitation data (mm, blue bars) and photosynthetically active radiation (PAR in μmol m^−2^s^−1^, black rectangles) for the measurement period. Weather data from the on-site weather station (**Figure 1**). Unmanned aerial vehicles (UAV) flight dates marked in vertical dashed lines.

**Figure 8** shows the H^2^ values of the measurements carried out on different days (top) and the weather data for the UAV flights (bottom). Overall, H^2^ values generally increased from flowering at the end of May up to a peak on the 2018-07-04. The increase in H^2^ values coincided with the dry period with no rainfall between the 2018-06-14 and the 2017-07-02. H^2^ values ranged from 0.30 to 0.67 for the blending mode “disabled” and from 0.36 to 0.74 for the blending mode “average.” The CT elicited with the blending mode “average” showed higher H^2^ values on all measurement dates except on the 2018-06-04 and the 2018-06-20. The variance components of the heritability split into genotypic and residual variance showed the blending mode “average” reducing both variances (**Figure 8**). The impact was however, larger for the residual variance than the genotypic variance.

The “stay-green” genotypes (red data points **Figure 8**, top) also showed a similar increase in H^2^ values after flowering at the end of May until beginning of senescence, with an outlier on the 2018-06-16 where the PAR was low compared to the other UAV flights (**Figure 8**, bottom). H^2^ values elicited with blending mode “average” were generally also higher than the blending mode “disabled,” except for the 2018-06-20. The blending mode “average” also reduced the genotypic and residual variance components for the “stay-green” genotypes. Further results are reported on the CT measured with the blending mode set to “average,” due to the generally higher heritability.

### Canopy Temperature Correlation Across a Day and Dates

**Figure 9:**
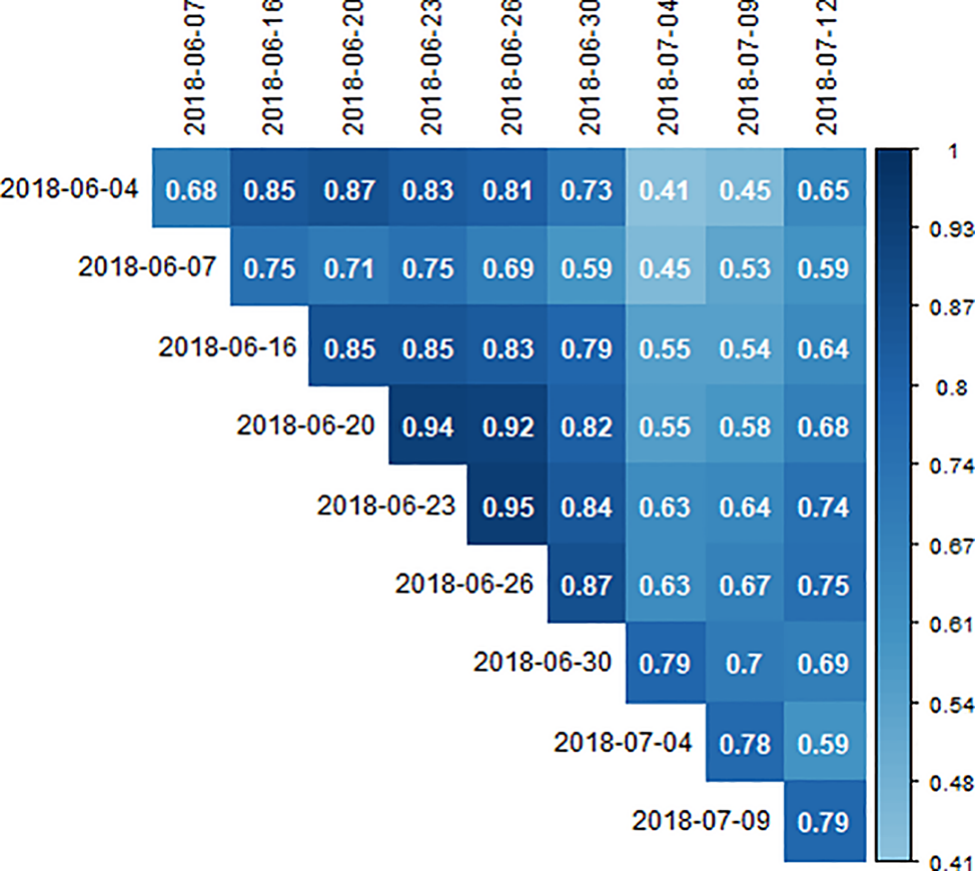
Correlation coefficients between spatially corrected genotypic canopy temperature (CT) values of different dates measured around solar noon. All correlations were significant at p ≤ 0.01.

**Figure 10:**
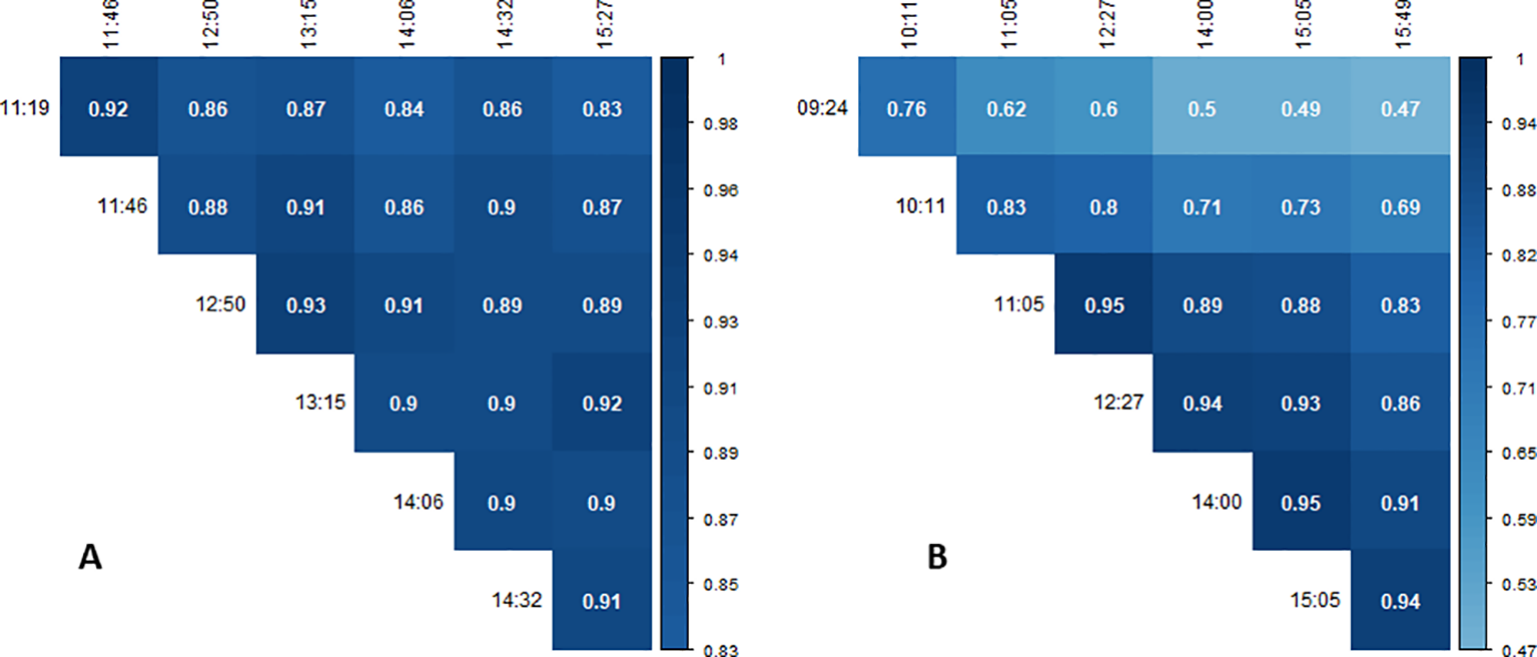
Correlation coefficients between spatially corrected genotypic canopy temperature (CT) values on the dates with multiple measurements: **(A)** the 2018-06-16 and **(B)** the 2018-06-20. All correlations were significant at p ≤ 0.01.

Correlation coefficients between the measurements performed around solar noon at the different dates ranged from 0.41 to 0.95 (**Figure 9**). All correlations shown in **Figure 9** were significant on p ≤ 0.01. Correlations between successive measurement dates were high and ranged between 0.68 and 0.95 (**Figure 9**, diagonal). Especially the three measurements between the period of the 2018-06-16 and the 2018-06-30 showed high correlations. For the 2018-06-16, Pearson correlations between measurements were overall high and ranged from 0.83 to 0.93 (**Figure 10A**). Correlations between successive flights were also high, ranging from 0.88 to 0.93. For the same-day measurements on the 2018-06-20, Pearson correlations ranged from 0.49 to 0.95 (**Figure 10B**). The two measurements conducted before solar noon (09:24 and 10:11 h) showed weak correlations with the measurements conducted around solar noon and the 15:05 and 15:49 h measurement. The solar noon measurements (11:05, 12:27, and 14:00 h) correlated highly.

## Discussion

This study presented a comparably cheap method for high throughput CT phenotyping based on UAV thermography in combination with photogrammetry and computer vision, namely the SfM approach. The total hardware cost added up to 18k € (6k € for the Matrice 600 pro, 8k € for the A65 camera, 3.5k € for Agisoft professional edition, 0.5k € controlling equipment). Additionally, a high-precision GNSS solution for measuring the GCP positions and a workstation for photogrammetric processing are also needed. With the flight parameters used in this study, an area of one hectare was captured in a flight time of approximately 8 min. Since the drone was powered by electricity, no substantial follow-up cost besides the cost of replacing the batteries occasionally needed to be considered. With increased flight time and increasing altitude, the approach potentially allows capturing very large areas since it allows combining many individual images to an orthomosaic. In the following, the results of this method are discussed.

### Orthomosaic Generation From Thermal Images

The orthomosaics showed high detail that allowed assessment of in-plot heterogeneity. Visually it can be seen that plot temperature is influenced by border effects of the between-plot space by approximately four pixels (approx. 0.25–0.3 m or two rows) (**Figure 5A, B**). Looking at the plots from multiple viewing geometries showed that CT is anisotropic. It shows an almost symmetrical pattern in parallel to the principal plane of the sun with on average several °C difference across the field of view (25°) of the sensor. Around nadir a hotspot is visible were the temperatures are slightly increased compared to the general pattern (**Figure 5D**). This can be explained by higher soil temperatures compared to plant temperatures, which are revealed when looking at very high-resolution images from the field phenotyping platform captured at the same time (**Figure 1**, **Supplementary Materials**). However, the effect of this differs depending on the canopy structure within each plot. Visual inspection of high-resolution images indicated that the angle of the heads and leafs also influence the apparent temperature.

The systematic differences resulting from the viewing geometry are also found in the blending modes. The blending mode “disabled” showed higher in-plot heterogeneity. In this blending mode, only the center part of each image is used, which corresponds to viewing geometries close to nadir (**Figure 5C**). This viewing geometry potentially captures more information from inside the canopy and the soil background than oblique viewing geometries (for a detailed discussion on the effects of the viewing geometry on the apparent signal—in particular the proportion of visible soil and plant material—please refer to Aasen and Bolten, 2018). In the blending mode “average” all information from all images covering a certain pixel in the orthomosaic is taken into account, and thus the information is averaged over a wide range of viewing geometries (including nadir and oblique). Consequently, compared to an only nadir viewing geometry, more information of plant material from the higher canopy levels is captured (Aasen and Bolten, 2018). This effect is also visible in the Bland-Altman plot (**Figure 6**). Negative differences between the blending mode “average” and “disabled” corresponds to higher apparent temperatures in the close to nadir viewing geometries (blending mode “disabled”). The negative slopes of the relationships indicate that with a higher absolute plot temperature the close to nadir values relatively increase. This can result from higher plot temperatures in less dense canopies (with lower biomass) were the nadir viewing geometry captures more of the warm soil background. Toward the later afternoon, the canopy cooling decreases and the soil background is shaded such that the nadir measurements become cooler (15:05 h flight) and later (15:49 h flight), the differences between the two measurements procedures become negligible. Similar, in the early morning (09:24 h flight) both soil and leaf temperature are largely determined by the air temperature, which result in negligible difference in apparent temperature between the blending modes. Overall, the interpretation of the results in this detail is very complex. More research is needed to disentangle the interaction of canopy structure, illumination, and viewing geometry with CT to establish a robust link between CT and actual physiological status (e.g., stomatal conductance) of the plants.

Many studies that use 2D imager [c.f. (Aasen et al., 2018)] based thermography have used single images for CT extraction (Bendig et al., 2012; Zarco-Tejada et al., 2012; Calderón et al., 2013; Deery et al., 2016; Sankaran et al., 2018; Deery et al., 2019), eliminating the need for complex image mosaicking. The drawback of the single image approach is that only a limited area can be captured—and to increase this area the flight height needs to be increased, consequently decreasing the GSD. Considering the limited resolution of current thermal cameras (most have a resolution of up to 640 x 480 pixels) and the limited maximum legal flying height of UAV systems in most countries, the applicability of the single image approach for low-cost UAV phenotyping is limited. Additionally, in the single image approach, anisotropy effects have a stronger influence on the data since a larger variety of viewing geometries are used within one image.

To achieve high position accuracy, GCPs are used during the generation of the orthomosaics (Ortega-Farías et al., 2016; Ribeiro-Gomes et al., 2017; Malbéteau et al., 2018; Sagan et al., 2019). A dense distribution of GCPs across the experimental site help to obtain optimal results (Mesas-Carrascosa et al., 2015; Roth et al., 2018). A key issue with conventional GCPs in thermal imagery is that they can be hard to detect in thermal images due to low contrast of such imagery (Malbéteau et al., 2018). This was confirmed in test flights conducted for this study. To overcome this limitation, some authors first georeferenced red green blue (RGB) images with GCPs and then referenced the thermal imagery to the RGB data (Sagan et al., 2019). With special thermal GCPs it is possible to georeference thermal orthomosaics without the need for exact for expensive on-board RTK solution for the UAV. Most studies did not report position accuracies of generated thermal orthomosaics (Berni et al., 2009b; Berni et al., 2009a; Zarco-Tejada et al., 2013; Maes et al., 2017; Santesteban et al., 2017; Malbéteau et al., 2018; Sankaran et al., 2018; Sagan et al., 2019). Ribeiro-Gomes et al. (2017) used rubber sheets with an aluminum plate as thermal GCPs and reported a thermal orthomosaic position RMSE of 7.2 m at a flight height of 80 m. After increasing the contrast of their thermal images, they reduced their position RMSE to 1.2 m. Malbéteau et al. (2018) used aluminum plates with black crosses taped onto but did not report spatial accuracies of their orthomosaics. Using regular GCPs, Gómez-Candón et al. (2016) report orthomosaic RMSEs ranging from 15 to 19.4 cm for their thermal flights at 40 m flight height. Compared to these studies, the obtained position accuracy in our study was very high, with positional RMSE ranging from 1.25 to 10.05 cm with an overall mean RMSE of 4.79 cm. Both Gómez-Candón et al. (2016) and Ribeiro-Gomes et al. (2017) used a thermal camera with similar resolution as in this study.

Some conclusions can be drawn from these results:

-When using the information of individual images or the blending mode “disabled” the flight pattern should be planned such that the plots are captured in similar viewing geometries since already small differences impact the apparent temperature. Ideally, the flight pattern should be along the range or rows of the design and with a high rate of frames per second.
-The “average” blending mode is able to reduce the impact of the viewing geometries. Since the anisotropy is symmetrically to the principal plane of the sun, a flight pattern in parallel to the principal plane is advised. Ideally, the capturing position is symmetrically in all directions, but it should at least be along the principal plane to appropriately average out the viewing effects. This would also be supported by a high measurement frequency.
-In case of slightly fluctuating measurement conditions, it is advised to fly in parallel to the range or row direction since differences can then be included into the range or row component of the spatial correction model. In future, models that integrate the measurement time could further improve the correction. Under strongly fluctuating environmental conditions it is not advised to measure, since the comparability of the measurements might be compromised due to the changes in plant physiology during the measurements.
-Independent of the data generation procedure, precise georeferencing is key if data from multiple flights should be processed in an automated way.

### Optimal Timing for Canopy Temperature Characterization

Comparing the genotypic CT values from multiple measurements on the 2018-06-16 and 2018-06-20, showed constant correlations across all measurement times on the 2018-06-16 (**Figure 10A**). On the other hand, the correlations of the morning measurements with subsequent measurements decreased toward the afternoon on the 2018-06-20 (**Figure 10B**). This indicated a changed response of the genotypes to the environmental conditions during the day with the rapid increase of temperature throughout the day on the 2018-06-20 compared to the moderate increase on the 2018-06-16 (21°C *vs*. 25°C span between the measurements).

H^2^ values were highest in the early afternoon at 14:00 h local time (**Figure 7**). This can be explained by the increased potential photosynthesis due to high irradiation and an increasing vapor pressure deficit toward the early afternoon (**Figure 7**), which can potentially increase conductance and may lead to an increased variance between the genotypes. These results align with the results of (Deery et al., 2016; Deery et al., 2019) for wheat. Thus, generally it can be concluded that flights at that time are best to estimate genotypic differences in CT. Still, the highest H^2^ on the 2018-06-16 was found around noon right after a cloud overpass. This could be an indication of differences between genotypes in upregulating transpiration after a cloud overpass during dry periods. Also, it is likely that the best timing depends on the water availability in the soil. Thus, to resolve such interactions, continuous measurements would be of benefit.

Looking at the whole period, H^2^ generally increased toward the beginning of July (**Figure 8**). The period between the 2018-06-14 and the 2017-07-02 corresponded to a dry period without rainfall (**Figure 8**, bottom) and it can be assumed that also the water stress increased in this period. Still, also the senescence started on the 2018-06-16 for the early senescent genotypes. In an attempt to disentangle the effect of phenology and water stress, we selected the “stay green” genotypes. These showed a similar trend in H^2^ as the whole-genotype set (**Figure 8**) suggesting that not only the senescence but also the ongoing dry period increased variance between genotypes. The drop in H^2^ observed on the 2018-06-16 for the “stay green” genotypes may have been a result of low PAR on that UAV flight, (**Figure 7**, left and **Figure 8**, bottom), possibly resulting in poor transpiration, which lowered the heritability and thus the ability to differentiate genotypes. Other flights on that day showed similar H^2^ as the flights on the 2018-06-20. This effect was not seen in the whole genotype set that contained already visual senescence genotypes. Still, it has to be noted that the “stay green” genotypes were only identified based on visual signs of senescence in this study, an pre-visual senescence processes might still influence the results. Consequently, to get stable CT estimates that correspond to physiology rather than phenology, the authors suggest performing CT measurements at early afternoon and before the onset of senescence. Further research is needed on this topic, since i) change in physiology during pre-visual senescence might already influence CT and ii) drought and phenology (i.e., early senescence) might interact and both have an effect on CT, as also mentioned in Lopes and Reynolds (2010).

### Comparison of Different Canopy Temperature Measurement Approaches

In a typical field phenotyping scenario, a couple of hundred to thousand plots situated on a few hectares need to be screened. For CT, several other measurement approaches exist besides the one described in this study. **Table 1** analyses the advantages and disadvantages of these approaches. Handheld measurements have a very attractive setup cost, are easy to setup and are highly flexible while arguably having the highest GSDs available. Sampling such a large field experiment by hand is in most cases unfeasible due to the high running costs, the long time needed to sample a plot and the changes in environmental conditions during the sample time, which result in low heritability (Deery et al., 2016; Sagan et al., 2019).

**Table 1:** Advantages (+, ++) and disadvantages (-, --) of the most common scenarios for eliciting canopy temperature (CT) in a field-phenotyping environment.

Property	Handheld sequential	Multiple devices simultaneous	Phenotyping platform sequential	Orthomosaic	Single image
Coverable area	--	-	+	++	0
Ground sampling distance	++	Non-imaging	++	+	0
				**UAV**	**Manned aircraft**
Setup cost	++	-	--	+	--
Running cost/field area	--	-	-	++	--
Setup effort	++	--	--	0	-
Effort for measurements	--	++	+	+	+
Plots sampled/time	--	++	0	+	++
Flexibility of sensor setup	++	-	--	++	+
Applicable for tall crops (>2 m)	-	--	0	++	++
Example publications	(Pask et al., 2012; Joalland et al., 2018)	(Jones et al., 2018; Deery et al., 2019)	(Kirchgessner et al., 2017)	(Sagan et al., 2019)	(Liebisch et al., 2015; Deery et al., 2016; Deery et al., 2019)

Since orthomosaics and single images can be captured by both unmanned aerial vehicles (UAVs) and manned aircrafts, thus in these columns it is separated by data product and platform aspects.

Phenotyping stations such as the “field phenotyping platform” (FIP) at ETH Zürich (Kirchgessner et al., 2017), the “Field Scanalyzer” in Rothamsted (Virlet et al., 2017) and similar stations described in Hund et al. (2019) are highly automated, reducing manual labor costs. Phenotyping stations have low effort for data acquisition, a moderately high area that they can efficiently cover and are applicable to measure tall crops to a certain extent (< 3 m). With the sensors situated only a few meters above ground, they have a very high GSD that allows differentiating differences between plant organs. However, they require high setup costs and are spatially very inflexible due to their stationary nature. In addition, the measurements are recorded sequentially, which might introduce biases due to changing environmental effects. Jones et al. (2018) and Deery et al. (2019) used a sensor network of infrared point sensors to simultaneously elicit CT for up to 84 out of 400 plots in their field experiments.

While this minimizes the running costs, acquiring a sufficient number of sensors to make such measurements viable requires high setup—and possibly maintenance—costs. However, such a system has the advantage that all measurements are done simultaneously, minimizing the impacts from changing environmental conditions during measurements.

**Table 1** contains the two scenarios “UAV orthomosaic” and “manned aircraft single image” for airborne measurements, whereas both—manned aircrafts and UAVs—could be used for both approaches. Still, these two scenarios are the most popular ones when it comes to airborne thermal field-phenotyping (Liebisch et al., 2015; Deery et al., 2016; Gómez-Candón et al., 2016; Malbéteau et al., 2018; Deery et al., 2019; Sagan et al., 2019).

The UAV has an advantage over manned aircrafts when it comes to setup and running costs of the measurement system. The effort for setup effort (sensor implementation) are roughly similar between the two systems. The size of the coverable area in an orthomosaic is only limited by the flight time of the carrier system but can be extended by combining imagery from multiple flights. The orthomosaic scenario can thus effectively cover a larger area than the single image approach at the cost of potential impacts of changing environmental conditions during the measurement of the images. The “single image” scenario, can sample more plots in a shorter time when the flight altitude is higher to capture all plots, at the cost of having a lower GSD than an orthomosaic captured from a lower altitude. Additionally, single images are limited in their covered area per image. The lower cost and administration needed to fly UAVs make this system more flexible than piloted aircraft.

A limitation of the UAV based orthomosaic employed in this study was the flying time of the multi-rotor UAV, which is currently at maximum 15 to 20 min. The high spatial resolution of the obtained orthomosaics meant that flight heights could potentially be doubled while still having a good GSD. Due to the high measurement speed of the camera, a higher flying speed would also be possible, extending the possible coverage per flight. The greatest benefit in sampling area could however be achieved by mounting thermal cameras on fixed-wing UAVs. Fixed-wing UAVs are able to cover large areas [up to tens of ha in one flight [e.g., (Wingtra, 2019; senseFly, 2019)].

## Conclusion

This study presented an unmanned aerial vehicle (UAV) based low cost thermal imaging approach to estimate canopy temperature (CT) for field phenotyping experiments. The approach allowed obtaining data with high temporal and spatial resolution at variable extents, since many thermal images can be mosaicked into one orthomosaic. Viewing geometry effects within the thermal imagery were analyzed and it was found that they potentially had large influences on the obtained signal within one image. It was discussed how these translated into effects in the thermal orthomosaic, depending on how the orthomosaics were generated. It was found that averaging the information of all images to characterize an area of interest (e.g., a plot) had a higher heritability that only using the center parts of the images during the mosaicking process. When averaging the information during the orthomosaic generation, it is suggested to use a regular grid of measurements in parallel to the principle plane of the sun and a high framerate. Correction for spatial effects in the data with the 2D splines of SpATS resulted in a heritability of 0.36 to 0.74 for CT measurements, depending on the day, flight time, and data processing mode. Analysis of multiple flights per day and across the season showed that an optimal time point for thermal measurements in wheat is before the onset of senescence and ideal flight times to estimate genotypic differences in CT are in the early afternoon around 14:00 h local time. Overall, the results of this study demonstrate that the low-altitude thermal remote sensing is suitable for high-throughput field phenotyping. A comparison to other approaches demonstrated that it helps to close the gap of existing applications of thermography in large-scale phenotyping scenarios for plant breeding. Future research should aim to establish a robust link between observed CT and plant physiological traits (e.g., stomatal conductance), since multiple results indicated a confounding effect of canopy structural traits such as canopy density, leaf, and head inclination.

## Data Availability Statement

The datasets generated for this study are available on request to the corresponding author. 

## Author Contributions

GP wrote large parts of the manuscript, did the post-processing of the thermal data/implementation of the processing chain, the statistical analyses, created the figures, build the GCPs and helped with the drone flights. AH set up and managed the field experiment (experimental design), contributed significantly to the manuscript and provided the code for the spatial correction. JA did the senescence scorings and contributed to the manuscript. LR supported the assembly and operation of the sensor/drone pack, gathering and pre-processing of weather station data, data processing pipeline and contributed to the corresponding part of the manuscript. MB supported the application and understanding of the SpATS methodology and corresponding R package and edited the corresponding parts of the manuscript. AW contributed by implementing the experiments within the Crop Science group at ETH and providing valuable feedback while writing the manuscript. FL supported conducting the experiment and writing the manuscript. HA conceived and set up the idea for the manuscript, contributed large parts of the manuscript, contributed largely to revisions, build the sensing system, performed the drone flights, developed the processing chain and supervised the study.

## Funding

We acknowledge support from the Swiss National Science Foundation under the “Phenocool” Project (Grant No. 169542). LR acknowledges funding from Innosuisse (www.innosuisse.ch) in the framework of the project ‘Trait spotting' (Grant No. KTI P-Nr 27059.2 PFLS-LS).

## Conflict of Interest

The authors declare that the research was conducted in the absence of any commercial or financial relationships that could be construed as a potential conflict of interest.
