# Supplementary material for: Assessment of Multi-Image Unmanned Aerial Vehicle Based High-Throughput Field Phenotyping of Canopy Temperature
Source: Front Plant Sci. 2020 Feb 25;11:150. doi: 10.3389/fpls.2020.00150 (PMC7052280; doi:10.3389/fpls.2020.00150)
Supplement: Supplementary file 1 [file DataSheet_1.pdf]

Supplementary Material

1 UAV flights

Table 1: Flight dates of UAV measurements. The BBCH stages (Lancashire et al., 1991) give the approximate growth stage of winter wheat during the UAV flights.

Date	Time	Average BBCH Stage	Date	Time	Average BBCH Stage
2018-06-04	13:47	73	2018-06-20	11:05	81
2018-06-07	15:20	75	2018-06-20	12:27	81
2018-06-07	15:48	75	2018-06-20	14:00	81
2018-06-16	11:19	78	2018-06-20	15:05	81
2018-06-16	11:46	78	2018-06-20	15:49	81
2018-06-16	12:50	78	2018-06-23	15:09	82
2018-06-16	13:15	78	2018-06-26	10:35	83
2018-06-16	14:06	78	2018-06-26	13:49	83
2018-06-16	14:32	78	2018-06-30	11:01	85
2018-06-16	15:27	78	2018-07-04	13:35	87
2018-06-20	09:24	81	2018-07-09	13:15	92
2018-06-20	10:11	81	2018-07-12	14:30	92+

2 Orthomosaic accuracies

Table 2: Accuracy parameters of the UAV orthomosaics generated through the processing pipeline as described in the section 2.4 of the main manuscript.

Date	Time	Altitude (m)	Resolution (cm/px)	Total Error (cm)	Total Error (px)
2018-06-04	13:47	75.10	5.07	8.56	0.92
2018-06-07	15:20	88.90	5.05	7.69	0.11
2018-06-07	15:48	74.60	5.04	5.68	0.08
2018-06-16	11:19	75.70	5.10	4.18	0.08
2018-06-16	11:46	94.00	5.11	5.89	0.10
2018-06-16	12:50	90.00	4.90	3.69	0.11
2018-06-16	13:15	79.70	5.19	5.62	0.08
2018-06-16	14:06	104.00	5.68	9.34	0.08
2018-06-16	14:32	74.80	5.04	4.01	0.09
2018-06-16	15:27	75.40	5.08	2.39	0.12
2018-06-20	09:24	74.20	5.01	9.43	0.08
2018-06-20	10:11	73.20	4.93	7.00	0.08
2018-06-20	11:05	78.80	4.93	6.31	0.08
2018-06-20	12:28	97.70	4.91	4.65	0.09
2018-06-20	14:00	75.10	5.07	5.62	0.07
2018-06-20	15:05	106.00	5.94	8.45	0.08
2018-06-20	15:49	108.00	6.00	13.94	0.07
2018-06-23	15:09	87.30	4.89	6.23	0.13
2018-06-26	10:35	92.90	5.00	9.08	0.08
2018-06-26	13:49	112.00	6.08	15.41	0.07
2018-06-30	11:01	87.30	5.08	10.05	0.10
2018-07-04	13:35	89.30	5.11	7.67	0.13
2018-07-09	13:15	75.70	5.10	4.43	0.10
2018-07-12	14:30	75.60	5.05	3.86	0.12

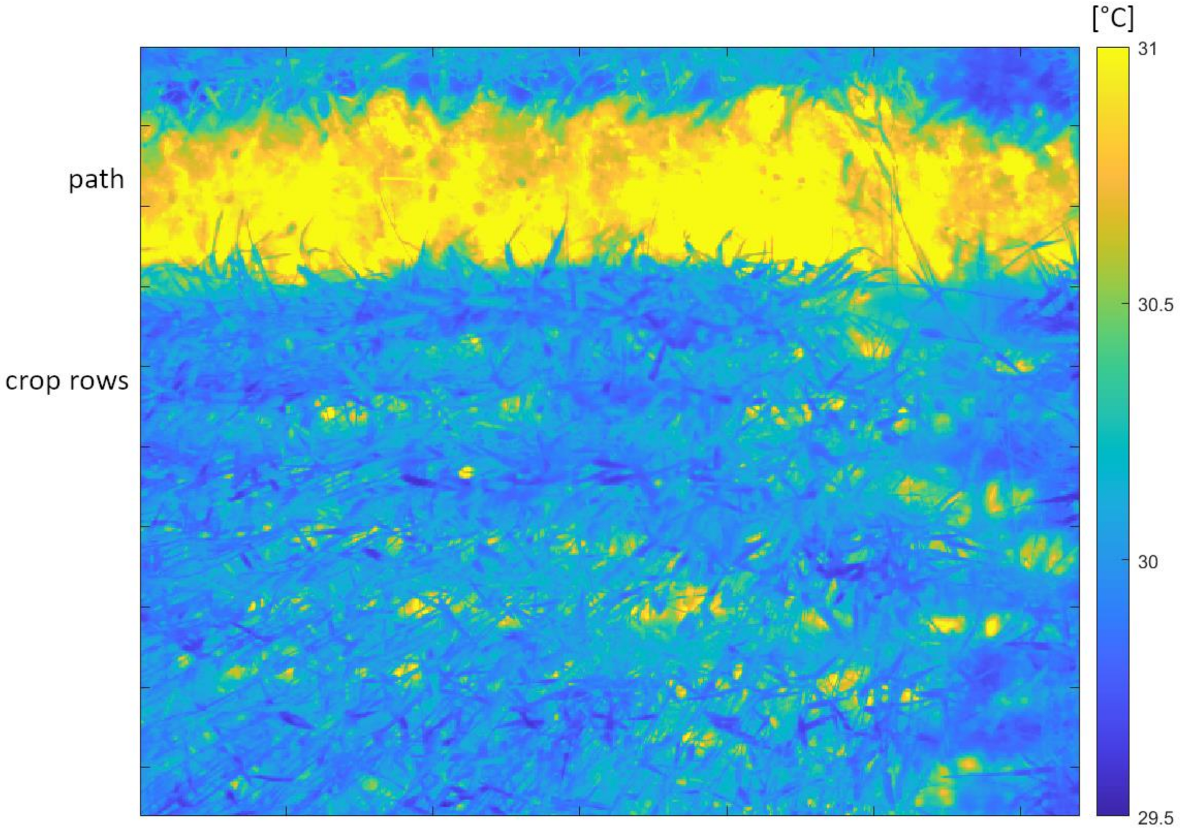

Appendix figure 1: High-resolution thermal image taken with the FIP phenotyping station (Kirchgessner et al. 2017) at nadir camera position taken at 11:39h on the 2018-06-20. The soil is considerably warmer than the canopy.

3 Linear relationship between blending modes

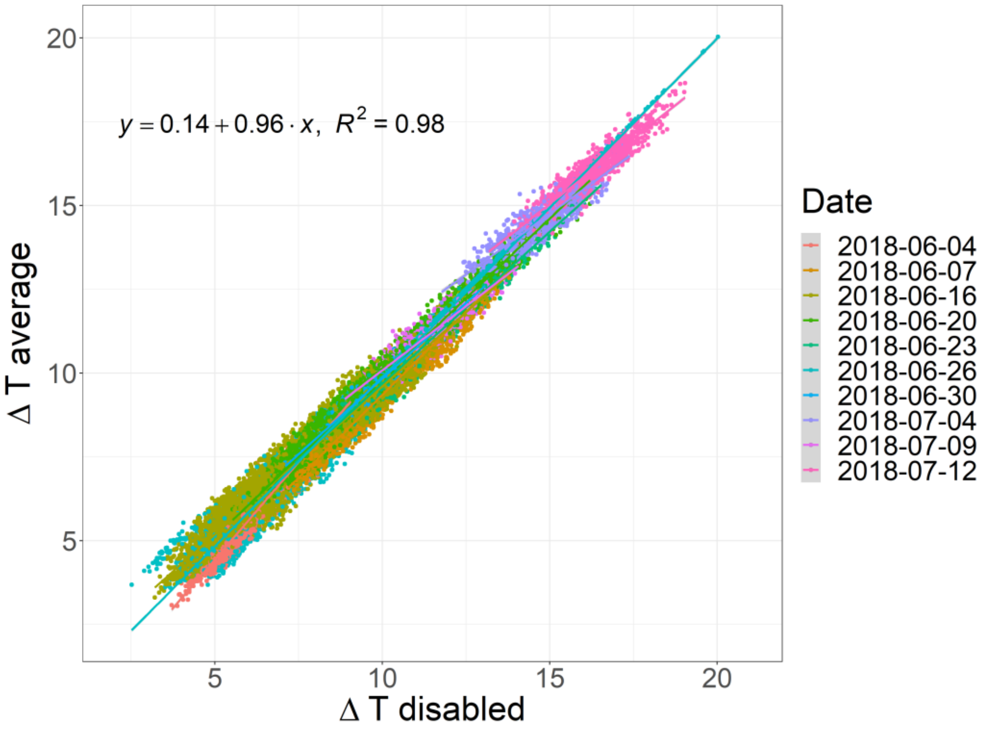

Appendix figure 2: Overall linear relationship (Intercept = 0.14, slope = 0.96, R^2 = 0.98) between the canopy temperature values obtained with the blending modes 'average' and 'disabled' colored by date of UAV flight.

Table 3: Parameters of the linear relation between the two evaluated blending modes ('average' and 'disabled' for each UAV flight (timestamp).

Timestamp	R2	Slope	Intercept
04.06.2018 13:47	0.92	1.16	-1.37
07.06.2018 15:20	0.90	0.94	-0.12
07.06.2018 15:48	0.78	0.81	1.58
16.06.2018 11:19	0.84	0.74	1.37
16.06.2018 11:46	0.87	0.88	0.64
16.06.2018 12:50	0.81	0.82	1.60
16.06.2018 13:15	0.88	0.80	1.37
16.06.2018 14:06	0.92	0.95	0.07
16.06.2018 14:32	0.93	0.91	1.11
16.06.2018 15:27	0.90	0.84	1.14
20.06.2018 09:24	1.00	1.00	0.00
20.06.2018 10:11	0.92	0.87	0.78
20.06.2018 11:05	0.96	0.89	0.84
20.06.2018 12:27	0.96	0.87	1.27
20.06.2018 14:00	0.98	0.92	0.81
20.06.2018 15:05	0.98	0.90	0.94
20.06.2018 15:49	1.00	1.00	0.00
23.06.2018 15:09	0.97	0.91	0.62
26.06.2018 10:35	0.73	0.70	1.50
26.06.2018 13:49	1.00	1.00	0.00
30.06.2018 11:01	0.96	0.90	0.81
04.07.2018 13:35	0.82	0.72	3.90
09.07.2018 13:15	0.87	0.77	2.43
12.07.2018 14:30	0.87	0.78	3.28
